# A novel method for prenylquinone profiling in plant tissues by ultra-high pressure liquid chromatography-mass spectrometry

**DOI:** 10.1186/1746-4811-7-23

**Published:** 2011-07-21

**Authors:** Jacopo Martinis, Felix Kessler, Gaetan Glauser

**Affiliations:** 1 Laboratory of Plant Physiology, University of Neuchâtel, 2000 Neuchâtel, Switzerland; 2 Chemical Analytical Service of the Swiss Plant Science Web, University of Neuchâtel, 2000 Neuchâtel, Switzerland

**Keywords:** Prenylquinones, ultra-high pressure liquid chromatography, quadrupole-time-of-flight mass spectrometry, light stress, *Arabidopsis thaliana*

## Abstract

**Background:**

Prenylquinones are key compounds of the thylakoid membranes in chloroplasts. To understand the mechanisms involved in the response of plants to changing conditions such as high light intensity, the comprehensive analysis of these apolar lipids is an essential but challenging step. Conventional methods are based on liquid chromatography coupled to ultraviolet and fluorescence detection of a single or limited number of prenylquinones at a time. Here we present an original and rapid approach using ultra-high pressure liquid chromatography-atmospheric pressure chemical ionization-quadrupole time-of-flight mass spectrometry (UHPLC-APCI-QTOFMS) for the simultaneous profiling of eleven prenylquinones in plant tissues, including α-tocopherol, phylloquinone, plastochromanol-8 and plastoquinone-9.

**Results and discussion:**

Mass spectrometry and chromatography parameters were optimized using pure standards. Sample preparation time was kept to minimum and different extraction solvents were evaluated for yield, ability to maintain the redox state of prenylquinones, and compatibility with chromatography. In addition to precise absolute quantification of 5 prenyllipids for which standards were available, relative quantification of 6 other related compounds was possible thanks to the high identification power of QTOFMS. Prenylquinone levels were measured in leaves of *Arabidopsis *grown under normal and high light intensities. Quantitatively, the obtained results were consistent with those reported in various previous studies, demonstrating that this new method can profile the full range of prenylquinones in a very short time.

**Conclusion:**

The new profiling method proves faster, more sensitive and can detect more prenylquinones than current methods based on measurements of selected compounds. It enables the extraction and analysis of twelve samples in only 1.5 h and may be applied to other plant species or cultivars.

## Background

**Figure 1:**
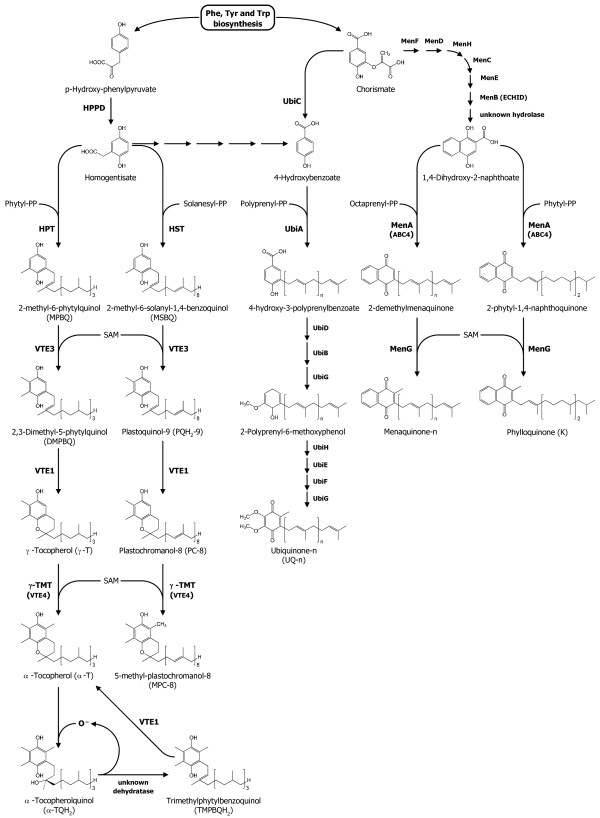
**Biosynthetic pathways of tocopherol, plastoquinol, plastochromanol, ubiquinone, and phylloquinone in *Arabidopsis***.

The exposure to high light (HL) intensities is a normal event for plants growing under field conditions and represents a source of stress to the photosynthetic apparatus, both by direct photodamage and because of the formation of reactive oxygen species (ROS). Higher plants have evolved an array of mechanisms in order to dissipate the excess energy and protect themselves from potential damage. In addition to state transition and changes in chloroplast ultrastructure, leaves acclimate to variations in light conditions through the accumulation of various antioxidants and the turnover of key photosystem components and reduced electron carriers. Prenylquinones, in particular, play a fundamental role in this process and include molecules with a broad action spectrum [1]. Plastoquinone-9 and ubiquinone-9, for example, are the two main lipophilic electron carriers in chloroplasts and mitochondria, respectively. In chloroplasts, α-tocopherol (vitamin E) acts as the major antioxidant together with the reduced form of plastoquinone-9 and its derivative plastochromanol-8, protecting membranes from photoxidative stress [2-8]. Phylloquinone (Vitamin K_1_), on the other hand, is also a strong antioxidant but its main biological role is as one-electron carrier in the A_1 _site of photosystem I. Moreover, it has been demonstrated that in cyanobacteria phylloquinone can be replaced by plastoquinone-9 in the active site, but this does not occur in higher plants [9-11]. There is increasing evidence that prenylquinone metabolic pathways closely intersect [12] (Figure 1) and thus a suitable analytical technique enabling the detection of global and subtle changes in the profile of these thylakoid lipids is required. Most of the current methods for the analysis of prenylquinones are based on their extraction by an organic solvent followed by chromatographic analysis of selected molecules. However, the solvent choice may represent a major limiting factor for the extraction and chromatography efficiency. In addition to their variable extractive power related to the polarity of each molecule, some of the commonly used protic solvents may promote the spontaneous oxidation of the reduced forms of some lipids. The extraction protocol can also influence the stability and the redox state of the extracted molecules: in particular, reduced forms can be easily oxidized by atmospheric oxygen if samples are exposed to air or to high temperatures for a prolonged period of time. The separation and detection of the components of thylakoid membranes represents another key factor to obtain unambiguous lipid profiles. Recent methods are based on normal or reverse-phase high performance liquid chromatography (HPLC) coupled to ultraviolet and fluorescence detection [12-14]. These techniques require long running times (several dozens of minutes) to obtain sufficient resolution and the identification of lipid constituents relies on the availability of pure standards. Mass spectrometry, despite its high potential for the detection and identification of various apolar lipids [15], has been scarcely employed in combination with liquid chromatography for the analysis of prenyllipids in plants.

In this paper we propose a novel method for prenylquinone analysis using an optimized sample preparation procedure followed by ultra-high pressure liquid chromatography-atmospheric pressure chemical ionization-quadrupole time of flight mass spectrometry (UHPLC-APCI-QTOFMS). Compared to conventional HPLC, UHPLC uses sub-2 μm particle supports which allows for higher efficiency and optimal velocity [16,17]. Consequently, high throughput separations can be obtained by reducing column lengths and increased flow rates. The QTOF mass spectrometer is particularly well adapted to coupling with UHPLC thanks to its rapid scanning rate [18]. Moreover its high mass accuracy gives access to the determination of molecular formula, an essential feature for reliable compound identification. Different solvents were tested for their extraction yield, their ability to maintain the redox state of molecules as well as their compatibility with reverse-phase UHPLC as injection solvent. Chromatography and mass spectrometry parameters were optimized for speed, selectivity and sensitivity. The applicability of the developed method was illustrated with the simultaneous quantification of several prenylquinones in *Arabidopsis thaliana *grown under normal and high light conditions.

## Results and Discussion

### Optimization of MS conditions

While there have been several publications on the LC-MS analysis of tocopherols [19,20] and vitamin K homologues [21,22] as they are essential vitamins for human metabolism, we are not aware of any report for plastoquinone-9 (PQ-9) and plastochromanol-8 (PC-8). Thus different QTOFMS parameters were evaluated to obtain maximal sensitivity for these latter molecules together with α-tocopherol (α-T) and phylloquinone (K). Electrospray and APCI were compared in both positive and negative ionization modes using standard solutions at 1 μg/mL. For PQ-9, the oxidized form (PQ) was used as standard. APCI was found largely superior to electrospray for all four compounds. K, PQ and PC-8 gave similar responses in positive and negative APCI but α-T was much better ionized in negative mode (ca. 8-fold), which was thus selected for further experiments. We then tested the effect of the probe temperature on the ionization efficiency. While α-T and K gave a higher signal at lower temperatures (350-500°C), PC-8 and PQ were best ionized at higher temperatures (475-600°C). A temperature of 475°C was selected as the best compromise for the detection of the four compounds. The source cone voltage was varied from 15 to 50 V and the highest response was obtained at 40 V. Other source parameters such as corona current (18 μA), source temperature (120°C) and desolvation gas flow (800 L/hr) had a less important effect on the MS signal. The influence of the mobile phase composition on the ionization efficiency was evaluated. Methanol (MeOH), acetonitrile (ACN) and tetrahydrofuran (THF) were compared and MeOH gave the best signal for all compounds. For α-T and K, the intensity of MS responses in MeOH was 2-5 fold higher than in ACN or THF. For PQ and PC-8 it was 10-40 fold higher. MeOH was thus selected as the organic solvent of choice for further LC method development.

### Optimization of LC conditions

**Figure 2:**
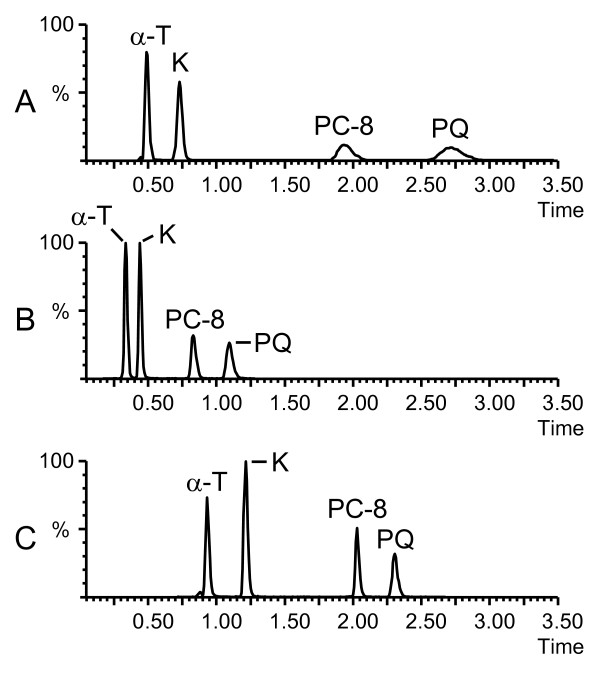
**Optimization of the chromatographic separation using pure standards**. A. Isocratic MeOH 100% as a mobile phase, T = 25°C. B. Isocratic MeOH 100% as a mobile phase, T = 60°C. C. Optimized gradient H_2_O/MeOH (10:90, v/v) - MeOH 100% in 1.5 min, followed by MeOH 100% for 2.5 min, T = 60°C. Other conditions are given in Materials and Methods. α-T, α-tocopherol; K, phylloquinone; PC-8, plastochromanol-8; PQ: plastoquinone-9 (oxidized form).

Prenyllipids are traditionally separated by isocratic normal-phase chromatography using diol columns and very apolar solvents or by reverse-phase (RP) chromatography using octadecyl (C18) columns and mixtures of MeOH/Ethanol or ACN/Ethanol as mobile phases. In RP mode, small amounts of hexane are sometimes used to accelerate the elution of PQ and PC-8 which are more hydrophobic than α-T and K. Run times are quite long (typically 20-40 min) because complete resolution of the peaks is needed for accurate quantification by ultraviolet and fluorescence detection. Using MS as a detector however, resolution becomes less important and chromatographic separations can often be shortened. In this study, UHPLC coupled to QTOFMS detection was evaluated for the high throughput analysis of prenyllipids. A 50 × 2.1 mm C18 column filled with 1.7 μm particles was selected for the separation. We first tested an isocratic 100% MeOH mobile phase at 25°C. Adequate retention factors (k) were obtained for α-T (k = 2.3) and K (k = 3.9), but PC-8 (k = 11.7) and PQ (k = 17) were too strongly retained leading to broad peaks and a decrease in sensitivity (Figure 2A). Since the use of MeOH as organic solvent was a required condition for MS sensitivity, we could not replace it by a solvent of higher eluting power. An alternative option to decrease the retention of PC-8 and PQ was to increase the temperature of the column. At 60°C, the retention factors for PC-8 and PQ were reduced to appropriate values, respectively 4.5 and 6.3. Meanwhile, the retention for α-T and K became slightly too weak with k values of 1.1 and 1.9 respectively (Figure 2B). To overcome this issue, a 1.5 min gradient from H_2_O/MeOH (10:90, v/v) to 100% MeOH was implemented at the beginning of the run followed by a hold at 100% MeOH for 2.5 min. Using this optimized separation, all compounds of interest eluted between 0.9 and 2.5 min and gave symmetrical and sharp peak shapes (Figure 2C). We verified that no thermal degradation occurred at 60°C. Although prenyllipids are neutral molecules and their retention times are not likely to be affected by a possible change in pH, we also tested the impact of mobile phase additives such as formic acid in terms of retention and ionization efficiency. As expected, no change in retention times was observed. On the contrary, the ionization efficiency was reduced by one order of magnitude for K and PQ, and more than two orders of magnitude for α-T and PC-8. This is not surprising since strong gas phase acids suppress proton transfers in negative APCI. It was found that even the injection of an acidic solution of standards could slightly reduce the ionization efficiency of α-T even if mobiles phases without additive were used. For this reason it is recommended to remove any trace of formic acid in the system, including the injector wash lines (if partial loop mode is employed), before prenyllipids are analyzed. Acids are also frequently used for lipid extractions in order to inhibit enzymatic hydrolysis. In the case of prenyllipids, this should be avoided.

### Detection of prenylquinones in plant samples

To evaluate the performance of the developed UHPLC-APCI-QTOFMS method on biological samples, we extracted *Arabidopsis *plants grown under normal and high light conditions in a mixture of chloroform/methanol (CHCl_3_/MeOH, 30:70, v/v). α-T, K, PC-8, and the oxidized (PQ) and reduced (PQH_2_) forms of PQ-9 were easily detected. The presence of PQH_2 _was confirmed by reducing the PQ standard with sodium borohydride and analyzing the resulting mixture. For these 5 molecules, positive identification was achieved based on the comparison of retention times and accurate masses with pure available standards. In addition, the QTOFMS allowed for the tentative identification of six other prenyllipids for which no standard was available, namely γ-tocopherol (γ-T), α-tocopherol quinone (α-TQ), hydroxyplastochromanol (PC-OH), hydroxyplastoquinone (PQ-OH) and the oxidized (UQ) and reduced (UQH_2_) forms of ubiquinone-9 (UQ-9). UHPLC-APCI-QTOFMS data acquired in negative and positive APCI for the identified prenyllipids are presented in Table 1.

**Table 1:** Prenylquinones identified from UHPLC-APCI-QTOFMS data acquired in negative and positive ionization modes.

No	RT (min)	APCI -	m/z (-)	APCI +	m/z (+)	formula	MS/MS (-)	Identification
1	0.76	(M)^-^	446.3763	-^a^	-^a^	C_29_H_50_O_3_	177.0919, 163.0764	α-tocopherol-quinone

2	0.92	(M-H)^-^	415.3575	-^a^	-^a^	C_28_H_48_O_2_	149.0605, 121.0655	γ-tocopherol

3	1.00	(M-H)^-^	429.3734	(M+H)^+^	431.3877	C_29_H_50_O_2_	163.0760, 135.0812	α-tocopherol

4	1.28	(M)^-^	450.3504	(M+H)^+^	451.3572	C_31_H_46_O_2_	210.0680, 185.0607	phylloquinone

5	1.52	(M-H^)-^	765.6183	-^a^	-^a^	C_53_H_81_O_3_	750.5956, 149.0608	hydroxyplastochromanol

6	1.71	(M)^-^	764.6102	-^a^	-^a^	C_53_H_80_O_3_	149.0604	hydroxyplastoquinone

7	1.72	(M-2H)^-^	748.6158	(M+H)^+^	751.6385	C_53_H_82_O_2_	188.0836, 149.0605	plastoquinol

8	1.89	(M-2H)^-^	794.6214	(M+H)^+^	797.6448	C_54_H_84_O_4_	779.5970, 219.0661	ubiquinol

9	2.12	(M-H)^-^	749.6227	(M+H)^+^	751.6395	C_53_H_82_O_2_	734.6009, 149.0608	plastochromanol-8

10	2.16	(M)^-^	794.6212	(M+H)^+^	795.6300	C_54_H_82_O_4_	779.5974, 219.0655	ubiquinone

11	2.43	(M)^-^	748.6152	(M+H)^+^	749.6232	C_53_H_80_O_2_	188.0841, 149.0606	plastoquinone

^a^, not detected. RT, retention time. Known chemical structures are available in Figure 1.

### Optimization of sample preparation

**Figure 3:**
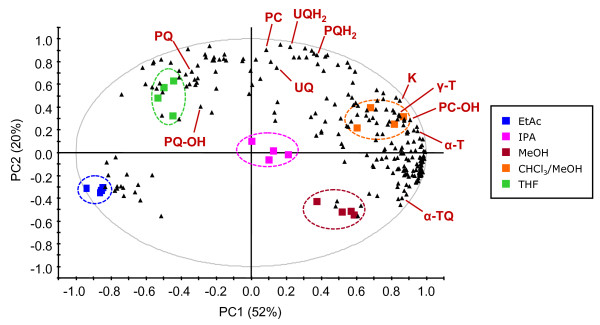
**Bi-plot derived from a principal component analysis (PCA) showing the selectivity of the extraction solvent**. Plants exposed to high light were pooled and extracted using the indicated solvents. Colored squares correspond to the observations (n = 4 for each solvent) and black triangles represent the variables. PC1 and PC2 are first and second principal components, respectively, with their percentage of explained variance. The identified prenyllipids are indicated (confront with Table 1). For abbreviations, see text.

**Figure 4:**
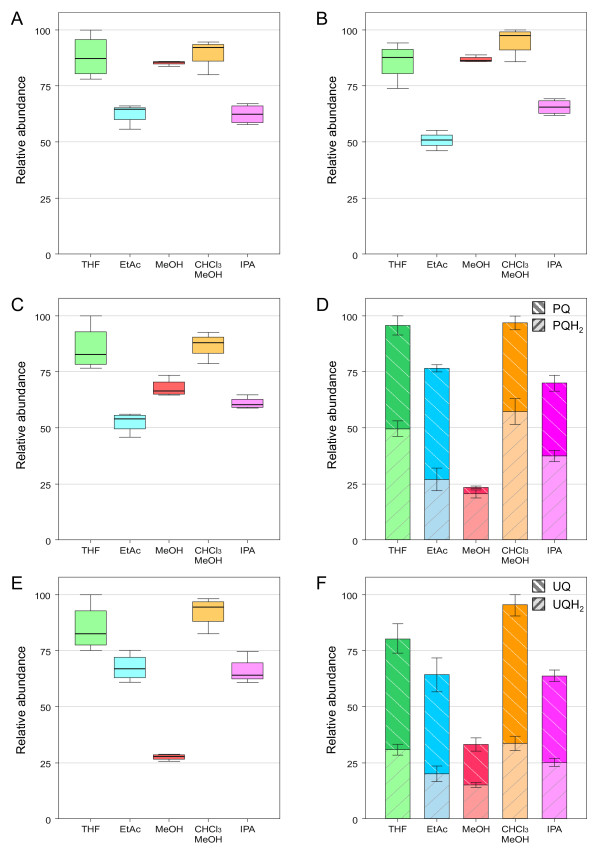
**Comparison of the extraction yields for prenylquinones with the five selected solvents**. A. γ-tocopherol. B. α-tocopherol. C. Phylloquinone. D. Plastoquinone-9, oxidized (PQ) and reduced (PQH_2_) forms. E. Plastochromanol-8. F. Ubiquinone-9, oxidized (UQ) and reduced (UQH_2_) forms. Plants exposed to high light were pooled and lipids were extracted using the indicated solvents. Data are from 4 replicates (± SD). For PQ-9 (D) and UQ-9 (F) the ability to maintain the redox state was also evaluated.

The above used extraction solvent (CHCl_3_/MeOH, 30:70 v/v) seemed to efficiently extract prenyllipids. However it had a serious drawback: CHCl_3 _is chemically poorly compatible with the UHPLC system used which can accommodate all common solvents except chlorinated solvents. We therefore evaluated different extraction solvents based on the three following criteria: extraction yield for the prenylquinones, ability to maintain the redox state of PQ/PQH_2 _and UQ/UQH_2_, and compatibility with UHPLC in terms of peak broadening and chemical resistance. A preliminary condition was to avoid an evaporation-redissolution step which would lengthen the sample preparation and possibly alter PQ/PQH_2 _and UQ/UQH_2 _redox ratios. Plants exposed to HL intensity (500 μE·m^-2^·s^-1^) were pooled and lipids were extracted using MeOH, isopropanol (IPA), ethylacetate (EtAC), THF and CHCl_3_/MeOH (30:70, v/v) as control and further analyzed by UHPLC-APCI-QTOFMS (n = 4). A principal component analysis (PCA) was performed on the obtained data. Interestingly, the five solvents clustered separately (Figure 3) and showed a significant selectivity for different molecules. In general, polar lipids including α-TQ were extracted more efficiently using MeOH, while more apolar solvents showed a higher extractive power for apolar prenyllipids (e.g. PQ and UQ). For all prenylquinones, the highest extraction yield was obtained with CHCl_3_/MeOH (30:70, v/v) and THF, while it was lower for EtAc and IPA (Figure 4A-F). MeOH represented a separate case because of its higher polarity, resulting in poor ability to extract PQ-9 and PC-8. Therefore MeOH is not a suitable solvent for PQ-9 and PC-8 analysis (Figure 4D and 4E).

Spontaneous variations in PQ/PQH_2 _ratio under standard working conditions were determined in the five solvents using a purified PQ standard. No spontaneous reduction was detected (*data not shown*). Moreover, when spiking PQ in leaf extracts, no increase in PQH_2 _was observed, suggesting that the reducing agents present in plant tissues are not concentrated enough to promote reduction. However, when ascorbic acid (30 mM) or butyl hydroxytoluene (BHT, 0.05%) were added to PQ solutions prepared in the different solvents, a slight reduction of PQ was observed (about 3% and 1% of the original amount of PQ using ascorbic acid or BHT, respectively). This phenomenon has previously been observed [13]. While anti-oxidant agents are often added to extraction experiments to prevent lipid peroxidation, this procedure does not appear useful for the determination of the redox state of PQ since reduction of the oxidized form may occur. When the PQ standard was chemically reduced with sodium borohydride and then exposed to air at room temperature, an increase of the oxidized form was detected for all solvents starting from 5-7 h after reduction. As a consequence, for the solvent to be effective in maintaining the original PQ/PQH_2 _ratio, it needs to oxidize the PQH_2 _present in plant tissues as little as possible. Using CHCl_3_/MeOH (30:70, v/v), IPA and THF, a PQH_2_/PQ_TOT _ratio ≥ 0.5 was found in plants exposed to HL conditions, while EtAc led to a PQH_2_/PQ_TOT _ratio of only 0.3 (Figure 4D). This may be due to lower extraction yield for PQH_2 _in EtAc rather than oxidation of PQH_2 _into PQ. Interestingly, when plants grown under normal light intensity (150 μE·m^-2^·s^-1^) were extracted in IPA, a complete oxidation of PQH_2 _was observed, while this did not happen in samples extracted by CHCl_3_/MeOH (30:70, v/v) and THF (*data not shown*). This may be explained by the higher amount of antioxidants (tocopherols or other lipid-soluble molecules) naturally present in thylakoid membranes under HL conditions, which could have protected PQH_2 _from spontaneous oxidation in IPA. A similar profile was observed for UQ-9 (Figure 4F).

**Figure 5:**
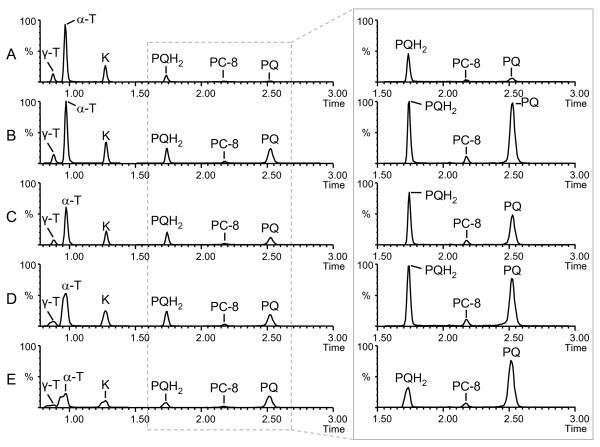
**Extracted ion chromatograms illustrating the influence of the five extraction solvents on the chromatographic performance**. A. MeOH 100%. B. CHCl_3_/MeOH (30:70, v/v). C. IPA. D. THF. E. EtAc. Plants exposed to high light were pooled and extracted using the indicated solvents. 5 μL was injected. Inset on the right: zoom on the peaks corresponding to PQ/PQH_2 _and PC-8. γ-T, γ-tocopherol; α-T, α-tocopherol; K, phylloquinone; PC-8, plastochromanol-8; PQ: plastoquinone-9 (oxidized); PQH_2_: plastoquinone-9 (reduced).

The influence of the extraction solvents on the chromatographic performance was also evaluated. Indeed, it is well-known that injection solvents of higher elution strength than the initial mobile phase can lead to peak distortion and broadening, in particular for early eluting peaks. No significant peak broadening was observed for MeOH, CHCl_3_/MeOH (30:70, v/v) and IPA (Figure 5A-C). Using THF, which is a stronger solvent, γ-T, α-T and K exhibited moderate peak broadening (Figure 5D). Not surprisingly, EtAc was the most problematic solvent: strong peak distortion was observed for γ-T, α-T and K and even PQH_2 _was affected (Figure 5E). We then diminished the injection volume from 5 to 2.5 μL, which substantially reduced peak broadening for THF but only slightly for EtAc (*data not shown*). In conclusion, 2.5 μL injections of solvents equally or more polar than THF could be used without negative effect on the chromatographic performance under the used conditions.

Overall, THF represented a good alternative to CHCl_3_/MeOH (30:70, v/v) since it provided high and reproducible extraction yields, best maintained the redox state of PQ-9 and UQ-9, and was chromatographically compatible. It was thus selected as the solvent of choice for the extraction of biological samples. To determine whether the volume of extraction solvent (500 μL THF for 100 mg of fresh leaf material) was sufficient for extracting most of the prenylquinones, we extracted leaves exposed to HL in 1500 μL or 500 μL (for the latter, the extract was further diluted three times) and extraction yields were compared (n = 3). No significant difference was found, confirming that a volume of 500 μL is sufficient for the extraction of 100 mg of fresh leaves. We noticed that the oxidation of PQH_2 _became significant about 2 and 5 hours after extraction for plants grown under normal and high light conditions respectively. For this reason, we took good care to always prepare and analyze samples within a maximum period of 1.5 hours. Given the speed of both extraction and analysis, 12 samples can be processed during that period of time. By performing sample preparation and analyses in parallel, a throughput of 100 samples in 8 h could be potentially achieved.

### Extraction recovery, matrix effects and limits of quantification

To evaluate sample preparation recovery, two experiments were carried out: first pure standards of α-T, K, PC and PQ were submitted to the extraction procedure. Recovery greater than 95% was obtained for all molecules. To determine if the plant matrix had an impact on the recovery, decyl-plastoquinone was used as non-endogenous structural analogue and spiked before and after extraction of plant samples at identical concentration. Again a recovery greater than 95% was obtained.

APCI is usually less prone to matrix effects than electrospray because ionization occurs in the gas phase. We nevertheless checked if prenyllipids were subjected to suppression or enhancement effects from the *Arabidopsis *extract. Since these molecules are endogenous in *Arabidopsis*, a THF extract was prepared and an aliquot was spiked with standard solutions of each prenyllipid. The control extract (C), the standard solutions (SS) and the spiked extract (SE) were injected and the obtained area compared. For all the ions, the area of C+SS were equivalent to those of SE with a variation inferior to 5%. In other words, no significant matrix effect was observed for the analyzed compounds.

The quantification of prenyllipids was based on internal standard calibration. Decyl-plastoquinone was found suitable as an internal standard: it was structurally close to the studied prenylquinones, was readily detected in negative APCI with high selectivity (M^- ^ion at *m/z *276.2087), eluted within the chromatographic gradient (retention time 0.44 min), and no matrix effect was observed. For α-T, PQ and PC-8, five calibrations points were used (0.05, 0.2, 1.0, 2.0, and 10.0 μg/mL for α-T; 0.05, 0.2, 1.0, 2.0, and 5.0 μg/mL for PQ-9 and PC-8). When PQ was completely reduced using sodium borohydride, the peak corresponding to PQH_2 _had an identical area to that of PQ. As a result, PQH_2 _could be quantified based on PQ calibration curve. For K, whose concentration does not significantly change after HL treatment, only four calibration points were used (0.1, 0.25, 1.0, and 2.5 μg/mL). For all compounds the response was linear over the range of the chosen concentrations with coefficients of determination > 0.99. A signal-to-noise ratio (s/n) of 10 was defined as limit of quantification (LOQ). For α-T, K and PC-8, the LOQ was 20 ng/mL. For PQ-9, an LOQ of 10 ng/mL was attained. The other identified prenyllipids (see Table 1) for which no pure standard was available were relatively quantified.

### Effect of high light on prenylquinone profile

**Figure 6:**
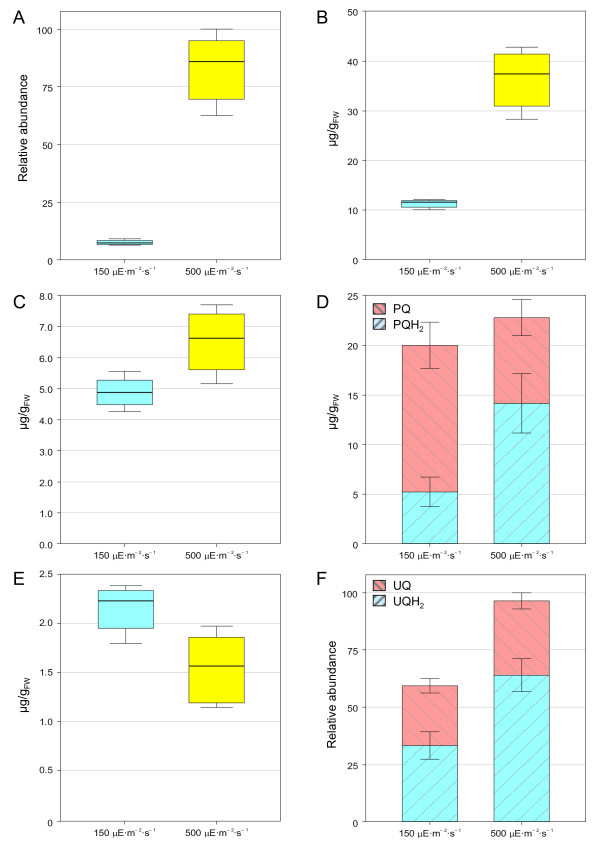
**Effect of HL exposure on the total leaf content of prenylquinones**. A. γ-tocopherol. B. α-tocopherol. C. Phylloquinone. D. Plastoquinone-9, oxidized (PQ) and reduced (PQH_2_) forms. E. Plastochromanol-8. F. Ubiquinone-9, oxidized (UQ) and reduced (UQH_2_) forms. Plants exposed to normal (150 μE × m^-2 ^× s^-1^) or high (500 μE × m^-2 ^× s^-1^) light intensities were extracted using THF. Data are from 4 biological replicates (± SD). For PQ-9 (D) and UQ-9 (F) the redox state of the total leaf pool was taken into account.

HL exposure induces biochemical and physiological changes in plants. In order to provoke a strong effect on prenyllipid content, 4 to 5-week-old *Arabidopsis thaliana *(Columbia-0) plants were exposed to continuous HL intensity for a prolonged period of time (7 days) and their prenyllipid profiles were compared to those obtained from plants grown under normal light. Under HL conditions γ- and α-tocopherol levels increased 8-fold and 3.5-fold respectively (Figure 6A and 6B). The synthesis of tocopherols and their accumulation in thylakoid membranes and plastoglobules is a well-known phenomenon which has been demonstrated to occur in response to oxidative stress [4,5]. After exposure to HL conditions plants produce more α-tocopherol, which mitigates photosystem II photoinactivation and protects thylakoids from photooxidative damage under chilling conditions [6,23]. No significant variation in phylloquinone levels was observed under HL (Figure 6C). As a matter of fact, even though its absence in plants lacking key enzymes in vitamin K biosynthetic pathway affects photosystem I activity [9], no direct evidence of its accumulation under HL has been reported [4]. In the future, we will apply this new method to profile simultaneously several tocochromanols/prenylquinones and assess the roles of each of them under various conditions.

Concerning plastoquinone-9, 4-week-old *A. thaliana *plants grown under normal light conditions showed a PQH_2_/PQ_TOT _ratio of about 0.25, in agreement with a previous study by Szymanska *et al*. [24] that used plants of similar age and a different method for prenyllipids extraction. After continuous HL exposure, plastoquinone-9 total content (oxidized + reduced) did not seem to be significantly altered. Yet, the redox state of the electron acceptor pool changed, with a significant accumulation of the reduced form (PQH_2_), leading to a PQH_2_/PQ_TOT _ratio of 0.6 (Figure 6D). This result suggests that the accumulation of PQH_2 _under HL may not be due to the *de novo *synthesis of the latter but on the reduction of the already available PQ pool. While these findings are distinct from the increase in PQH_2 _synthesis reported by Szymanska *et al*. [24], this discrepancy may well be attributed to the different growth and light conditions or to the different reference units employed (μg/g fresh weight versus μg/mg chlorophyll).

Among the other prenyllipids identified, plastochromanol-8 levels did not significantly change when plants were exposed to HL (Figure 6E), while total ubiquinone-9 content increased about 1.5-fold. Moreover, the UQH2/UQ_TOT _ratio increased in response to the change in light conditions (Figure 6F), as previously observed by Yoshida *et al*. [14].

## Conclusion

The presented method introduces for the first time the use of UHPLC-APCI-QTOFMS for simultaneously profiling several prenylquinones in plants. It proves to be fast, reliable, very selective and sensitive for the analyzed molecules, and consume less solvent than conventional methods. By combining it with simple and rapid sample preparation, a single plant can be extracted and analyzed in less than 15 min and twelve samples can be processed in 90 min. Moreover it allows for the detection and tentative identification of molecules for which no pure standard is available. The developed method will be used to profile prenylquinones in various *Arabidopsis *mutants as well as in other commercially relevant crop species.

## Methods

### Chemicals

The solvents used for extraction were methanol (MeOH, HPLC grade, Chromanorm), chloroform (CHCl_3_, analytical grade, Normapur) and tetrahydrofuran (THF, analytical grade, Normapur) from VWR (Leuven, Belgium), isopropanol (IPA, HPLC grade) and ethylacetate (EtAc, analytical grade) from Acros Organics (Geel, Belgium). ULC/MS grade MeOH and water from Biosolve (Valkenswaard, The Netherlands) were used for the UHPLC-APCI-QTOFMS analyses.

α-T and K standards of HPLC grade (≥ 99.5%) were purchased from Sigma-Aldrich (Steinheim, Germany). Decyl-plastoquinone (~75%) was obtained from Sigma-Aldrich. PQ-9 and PC-8 standards were provided by Jerzy Kruk (Jagiellonian University, Kraków, Poland). The oxidized and reduced PQ-9 standards were obtained as described in [25] with slight changes. Briefly, an excess (1 μg) of sodium borohydride (Fluka, Buchs, Switzerland) was added to the oxidized PQ standard (100 ng) to completely reduce it to PQH_2_. The retention time of both forms was then determined by UHPLC-APCI-QTOFMS. Ascorbic acid was purchased from Carl Roth (Karlsruhe, Germany) and butyl hydroxytoluene (BHT) from Sigma-Aldrich.

### Plant material and treatments

*Arabidopsis thaliana *(Columbia-0) plants were grown on soil under standard growth conditions (150 μE·m^-2^·s^-1^, 8/16 h light/dark period, 21/18°C, 55% relative air humidity) according to the protocol described in [26] with slight modifications. HL treatment was performed on 4- to 5-week-old rosettes by exposure to continuous HL conditions (500 μE·m^-2^·s^-1^, 21°C, 55% relative air humidity) for 1 week in a PGC 6HID growth chamber (Percival Scientific, Boone, IA) equipped with 400 W metal halide lamps (Philips).

### Prenyllipid extraction

*Arabidopsis *leaves from 4- to 5-week-old rosettes were ground in a mortar with liquid nitrogen. Approximately 100 mg of leaf material was then exactly weighed, transferred to a 1.5 mL microcentrifuge tube (Eppendorf, Hamburg, Germany) and swiftly re-suspended in five volumes of the selected solvent (e.g. 500 μL for 100 mg) containing decylplastoquinone at 2 μg/mL as internal standard. Care was taken that no thawing occurred before the solvent was added. Glass beads of about 1 mm of diameter (Assistent, Sontheim, Germany) were added and samples were further homogenized for 3 min at 30 Hz in a tissue lyser (Retsch MM 300, Haan, Germany). Tubes were centrifuged on a benchtop centrifuge (14,000 × g for 3 min at 4°C) and 400 μL of supernatant was then transferred to an appropriate glass vial for immediate UHPLC-QTOFMS analysis.

### Liquid chromatography-mass spectrometry analysis

The LC-MS system consisted of a Waters Acquity UPLC™ (Milford, MA) coupled to a Waters Synapt G2 MS QTOF equipped with an atmospheric pressure chemical ionization (APCI) source. Prenyllipids were separated on an Acquity BEH C18 column (50 × 2.1 mm, 1.7 μm) under the following conditions: Solvent A = water; Solvent B = MeOH; 90-100% B in 1.5 min, 100% B for 2.5 min, re-equilibration at 90% B for 0.5 min. The flow rate was 800 μL/min and the injection volume was 2.5 μL. The temperature of the column was set to 60°C and the autosampler chamber was kept at 15°C. Data were acquired with a scan time of 0.4 s over an *m/z *range of 225-1200 in the negative ion MS mode. The corona current was set to 18 μA and the cone voltage to 40 V. The source temperature was maintained at 120°C and the APCI probe temperature at 475°C. The desolvation gas flow was set to 800 L/hr. The mobile phase was diverted to waste for 0.3 min at the beginning of the gradient. Accurate mass measurements were obtained by infusing a 400 ng/mL solution of the small peptide leucin-enkephalin at a flow rate of 10 μL/min through the Lock Spray™ probe. For the identification of prenyllipids, positive and negative ion MS/MS experiments were carried out using a fixed collision energy of 40 eV and argon as collision gas at a flow of 2.1 mL/min. The quadrupole LM resolution was 4.7, and the HM resolution was 15. MS/MS product ion spectra were acquired over the *m/z *range 50-1200. Absolute quantities of prenyllipids were determined using standard curves obtained from standard compounds. The concentrations of the calibration points for α-T were 0.05, 0.2, 1.0, 2.0, and 10.0 μg/mL, for PQ-9 and PC-8 0.05, 0.2, 1.0, 2.0, and 5.0 μg/mL. For K, the concentrations were 0.1, 0.25, 1.0, and 2.5 μg/mL. All standard solutions contained decylplastoquinone (internal standard) at a concentration of 2 μg/mL.

### Data treatment

Data were processed using Masslynx v4.1 (Waters). Multivariate analysis was carried out using MarkerLynx XS™ (Waters). The following parameters were used: initial and final retention times 0.7-3.0 min, mass range *m/z *225-1200 Da, mass tolerance 0.03 Da, retention time window 0.10 min, automatic peak width detection, intensity threshold 1000 counts. The deisotope filtering function was applied. Non-normalized peak areas were generated. Variables were UV-scaled before applying PCA.

## Competing interests

The authors declare that they have no competing interests.

## Authors' contributions

JM grew, collected, and extracted plants. GG developed the UHPLC-APCI-QTOFMS method and conducted measurements. JM and GG carried out the optimization of the sample preparation, performed data analysis and treatment. GG and JM wrote the manuscript. FK contributed to the writing of the manuscript. All authors conceived the study. All authors read, commented and approved the final manuscript.
